# COVID-19's Impact on Italian Urology

**DOI:** 10.1590/S1677-5538.IBJU.2020.S103

**Published:** 2020-07-27

**Authors:** Francesco Esperto, Rocco Papalia, Ana María Autrán-Gómez, Roberto M. Scarpa

**Affiliations:** 1 University of Rome Campus Bio-medico Department of Urology Rome Italy Department of Urology, Campus Bio-medico, University of Rome, Rome, Italy; 2 University Hospital Fundación Jiménez Díaz Department of Urology Madrid Spain Department of Urology University Hospital Fundación Jiménez Díaz, Madrid, Spain

**Keywords:** COVID-19 [Supplementary Concept], Urology, Pandemics

## Abstract

The COVID-19 pandemic has impacted our lives, our habits and our healthcare system. Italy is one of the countries affected first and more aggressively from the outbreak. Our rapidity has been guide for other healthcare systems from around the World. We describe the impact of COVID-19 on Urology, how the Urological scientific community responded to the emergency and our experience in a high-volume Roman University hospital. The aim of our work is to share our experience providing suggestions for other global hospitals on how to manage the COVID-19 emergency.

## EPIDEMIOLOGY

The severe acute respiratory syndrome coronavirus 2 (SARS-CoV-2) was first identified in Wuhan, Hubei Province, China, in December 2019 ( 1 ). The disease has been termed COVID-19 and on March 11^th^ 2020, The World Health Organization (WHO) declared it as a pandemic ( 2 ). On April 21^st^, 2,585,468 COVID-19 positive cases and 178,854 deaths have been confirmed worldwide. The United States, Spain and Italy were the most affected countries with 825,306, 208,389 and 183,957 positive cases respectively ( 3 ).

## ITALY

**Figure 1:**
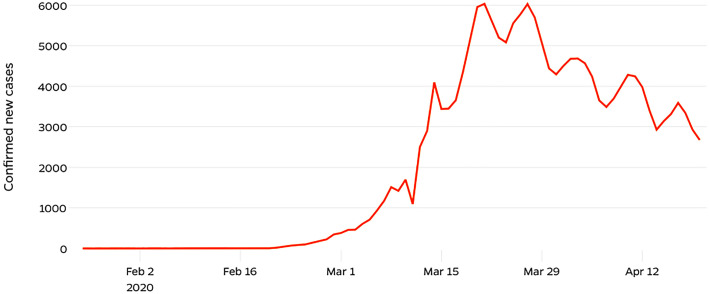
The outbreak evolution curve of confirmed COVID-19 new cases in Italy from Feb 2 to April 12 ( 5 ).

COVID-19 was first detected in Italy on January 30^th^, and the Italian government immediately declared the state of emergency. A COVID-19 task Force and a Special Commissioner for the Emergency were appointed. On February 23^th^, a ban was put on entry and exit in the municipalities where outbreaks occurred, and public events were suspended. National lock down was officially announced on March 9^th^ ( 4 ). The number of cases increased since the first case and peaked in mid-March ( Figure-1 ) ( 5 ).

**Figure 2:**
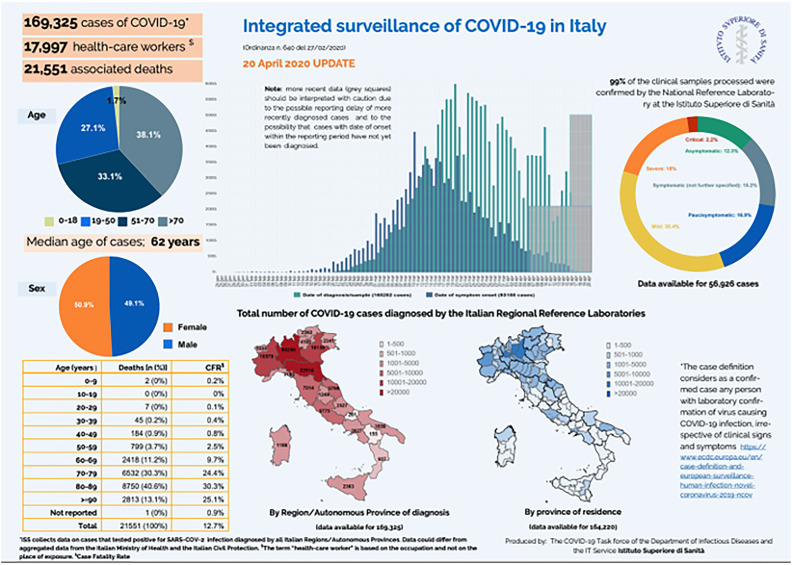
Integrated surveillance of COVID-19 in Italy ( 6 ).

**Figure 3:**
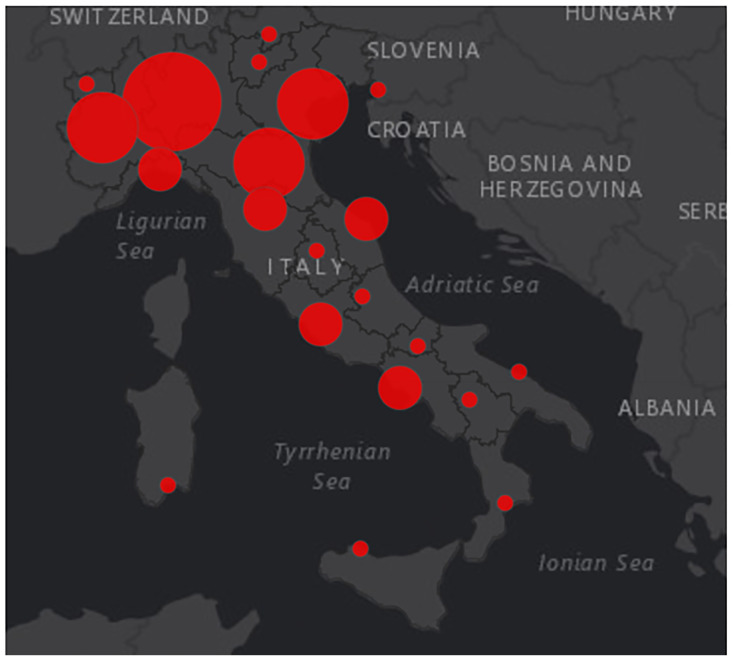
Distribution of COVID-19 cases in Italy ( 3 ).

On April 29^th^ the number of COVID-19 associated deaths was 26,977. A total of 17,997 healthcare workers were tested positive for COVID-19 (median age 48 years, 32% male), which accounted for 10.7% of the total number of reported cases. The high transmission potential of the virus in the healthcare sector was evident ( Figure-2 ) ( 6 ). Overall, 79,370 cases were male (50.0%) and the median age amongst both genders was 62 years (range 0-100). In the age groups 0-9, 10-19, 60-69 and 70-79 years, a greater number of male cases compared with female were observed. There was an increase in lethality with increasing age of cases. Lethality was higher in male subjects in all age groups, except for the age group >90 years. In 31.1% of the reported cases, at least one co-morbidity was identified (cardiovascular, respiratory, diabetes, immune deficiencies, metabolic, oncological, obesity, kidney and other chronic pathologies) ( 6 ). Presenting symptoms of COVID-19 patients included fever (75%), dyspnea (72%), cough (38%), diarrhea (6%), hemoptysis (1%). Overall, 60.7% of COVID-19 deaths were associated with 3 or more pre-existing diseases ( 4 ). The distribution of COVID-19 cases varies within the country. The north is much more affected and is reflected on the mortality rate ( Figure-3 ) ( 3 ).

At the 28^th^ of April the most affected regions were Lombardy, Piedmont, Emilia-Romagna, Veneto, Tuscany and Liguria with an amount of total cases detected of 74348, 25450, 24914, 17708, 9231, 7772 respectively. The mortality rate was the following: 18.3 % in Lombardy, 11.5 % in Piedmont, 13.9 % in Emilia Romagna, 7.95 % in Veneto, 8.8 % in Tuscany, 14.7 % in Liguria.

The less affected regions were Calabria, Basilicata and Molise with a mortality rate of 7.7 %, 6.8 %, 7.1 % respectively. Lazio with 6467 cases detected had a mortality rate of 6.4 %.

Intensive care units (ICUs) filled up quickly with COVID-19 patients and became stretched to accommodate non-COVID-19 patients who required critical care. In Lombardy Grasselli et al. described a mortality rate of 26% in ICUs as of March 25, 2020 ( 7 ). The initial lack of personal protective equipment (PPE) and swab testing led to a rapid spread. Healthcare professionals were being infected, reducing the number of healthcare workers (HWs) available to manage the emergency. However, the situation was different in less affected regions where there was more time to prepare and organize resources and HWs to manage the pandemic.

## MEASURES

Members of the RUN (Research Urology Network) group provided guidance on the management of urological patients during the COVID-19 era. They suggested treatment for urgent or emergent urological conditions only. Factors affecting the categorization of procedures included the need for postoperative intensive care, need for blood transfusion or other blood products, cardiovascular, respiratory or infective comorbidities, and the need for psychophysical support. Indications in postponing up to six months prostate biopsies, flexible cystoscopies, replacements of ureteral stents and nephrostomy tubes, intravesical therapy for low and intermediate risk bladder cancers have been provided ( Table-1 ) ( 8 ).

**Table 1:** Factors potentially affecting the choice of the different urological procedures during COVID- 19 pandemic (8).

Procedure	Indication for the emergency phase	Note
Prostate biopsy	Postpone	Reconsider performing prostate biopsy in patients with high clinical suspicion of prostate cancer if the emergency phase should prolong
Flexible cystoscopy	Postpone	Reconsider performing cystoscopy in patients with high risk bladder cancer if the emergency phase should prolong
Replacement of ureteral stents and nephrostomy tube	Postpone up to 6 months	
Intravesical therapy for high risk bladder cancer	Do not postpone	
Intravesical therapy for low or intermediate risk bladder cancer	Postpone	

The admission pathway included pre--admission telephone triage, nasopharyngeal swabs, PPE, strict rules in the operating room ( 9 ). Ribal et al. produced dedicated European Associations of Urology (EAU) guidelines on the management of Urological patients during the COVID-19 outbreak ( 10 ). Despite strong efforts in trying to prioritize oncological and urgent procedures, the burden of oncological patients on waiting list is increasing. Campi et al. described the progressive reduction in all elective's procedures in three high volume Urology centers in Italy ( 11 ).

### Impact on training

Resident's programs in Europe and in Italy are just harassed from a lack of adequate academic and surgical training ( 12 – 15 ) and it is known how its associated frustration may lead to burnout ( 16 ). Several papers described a global slowdown of Urology residence program during the pandemic ( 17 – 19 ). Social Media and smart learning implementation have been proposed as valid tools to supply scientific knowledge during this scenario ( 20 , 21 ). Our group asked ‘what should be the role of residents during a pandemic?'. On calling the Hippocrates statement (Afhprism 1,1) “Ὁ βίος βραχύς, ἡ δὲ τέχνη μακρή, ὁ δὲ καιρὸς ὀξύς, ἡ δὲ πεῖρα σφαλερή, ἡ δὲ κρίσις χαλεπή” “Vita brevis, ars longa, occasion praeceps, experimentum periculosum, iudicium difficile”, we think residents should play an active role alongside the specialist by exploiting the pandemic as an unrepeatable opportunity from which to learn upon ( 22 ). In general, the COVID-19 emergency is a highly dynamic situation and the burden on the healthcare system varies daily according to the geographical region.

### Our Experience

Campus Bio-Medico University is a high--volume university Hospital in Rome. It has no Accident and Emergency (A and E) Department and currently is COVID-19 free. We continue to operate on oncological and urgent patients with safety precautions. Information about the virus, local policies, patients' access to the hospital, surgery protocols and individual protection have been provided to all HWs ( Table-2 ). Every 8 hours a FFP2 mask is made available at the main entrance of the hospital. An open Outpatient COVID-19 Clinic (composed of 2 senior and 3 junior Internal Medicine Consultants) for HWs has been established to review those with symptoms or have been in close contact with a positive or suspect COVID-19 patient.

**Table 2:** Summary of the COVID-19 task force actions regarding PPE of Health workers (HWs).

Front office staff working		Healthcare personnel in contact with patients					Laboratory staff in contact with biological samples
At station in direct contact with patients	At station with protective glass	In contact with a suspected or confirmed case of COVID-19	In contact with a patient who presents symptoms of fever and / or cold and / or cough	Performing endoscopic procedures	Assigned to take a biological sample for COVID-19 + patient	Anesthesiologists performing intubation	
frequent hand hygiene by using 60% alcohol solution	frequent hand hygiene by using 60% alcohol solution	FFP2 filtering mask (use FFP3 only for the procedures that generate aerosols)	FFP2 filtering mask (use FFP3 only for the procedures that generate aerosols)	FFP3 filtering masks	FFP3 filtering mask	FFP3 filtering mask	FFP3 filtering mask
wear the FFP2 filtering mask during the entire work shift	/	goggles or visors to protect eyes from biological liquids ‘splashes	goggles or visors to protect eyes from biological liquids ‘splashes	goggles or visors to protect eyes from biological liquids ‘splashes	goggles or visors to protect eyes from biological liquids ‘splashes	goggles or visors to protect eyes from biological liquids ‘splashes	goggles or visors to protect eyes from biological liquids ‘splashes
wear protective glasses from liquids splashes during the entire work shift	/	water repellent PPE coat	/	water repellent PPE coat	water repellent PPE coat	water repellent PPE coat	water repellent PPE coat
provide a surgical mask, supplied at the desk, to be worn by the patient with visible respiratory symptoms	provide a surgical mask, supplied at the desk, to be worn by the patient with visible respiratory symptoms	double gloves	gloves	gloves	double gloves	double gloves	double gloves

Access to the Hospital is regulated through telephone triage to rule out any symptoms or suspicion of COVID-19. In suspected cases, responsible physicians would call to clarify. In non-suspected cases, patients could go for hospital admission as pre-planned. In suspected cases, patients are instructed to stay at home and call their GP for further advice. The access to the hospital is allowed from a unique entrance with security check.

A surgical facemask and hand hygiene with 60% alcoholic solution are provided to everyone at the entrance. Temperature is checked through a thermoscanner, symptoms are checked and reason for admission is evaluated. If no issues are encountered during this phase, patient can access the hospital. If any doubts are raised during the admission check, the responsible physician would review the case and decide whether to proceed with pre-planned admission or to refer to a dedicated COVID team.

We have detected three positive patients (in droplet isolation inside the hospital from the beginning of their admission) who have been transferred to COVID-19 centers within 48 hours. All the healthcare staff who had been in contact with them have been swabbed twice with negative results, showing the efficacy of the policy undertaken.

Visitors are allowed to access the hospital for a limited span of time (1 hour) and only one person per patient are allowed to visit after strict security checks performed at the main entrance.

We developed telemedicine protocols for outpatient's clinic and arranged virtual multidisciplinary meetings for oncological patients. Our surgical activity increased in volume, performing exclusively elective oncological and urgent operations. All patients treated have been called two weeks after discharge and none have declared any symptoms of COVID-19.

## CONCLUSION

COVID-19 emergency is a highly dynamic situation and the burden on the healthcare system varies daily according to the geographical region. Through meticulous hospital instructions, prompt adoption of PPE, controlled access to the hospital, and prompt management of suspected/positive cases, we were able to maintain a COVID-19 free hospital and to continue our surgical activities during the pandemic.
